# Emerging trends in virus and virus-like particle gene therapy delivery to the brain

**DOI:** 10.1016/j.omtn.2024.102280

**Published:** 2024-07-19

**Authors:** Heshadi Primrose Mandalawatta, K.C. Rajendra, Kirsten Fairfax, Alex W. Hewitt

**Affiliations:** 1 Menzies Institute for Medical Research, University of Tasmania, Hobart, TAS, Australia; 2 School of Medicine, University of Tasmania, Hobart, TAS, Australia

**Keywords:** MT: Delivery Strategies, neurological diseases, blood-brain barrier, gene editing, delivery systems, engineered virus-like particles, virus pseudotyping

## Abstract

Recent advances in gene therapy and gene-editing techniques offer the very real potential for successful treatment of neurological diseases. However, drug delivery constraints continue to impede viable therapeutic interventions targeting the brain due to its anatomical complexity and highly restrictive microvasculature that is impervious to many molecules. Realizing the therapeutic potential of gene-based therapies requires robust encapsulation and safe and efficient delivery to the target cells. Although viral vectors have been widely used for targeted delivery of gene-based therapies, drawbacks such as host genome integration, prolonged expression, undesired off-target mutations, and immunogenicity have led to the development of alternative strategies. Engineered virus-like particles (eVLPs) are an emerging, promising platform that can be engineered to achieve neurotropism through pseudotyping. This review outlines strategies to improve eVLP neurotropism for therapeutic brain delivery of gene-editing agents.

## Graphical abstract

**Figure:**
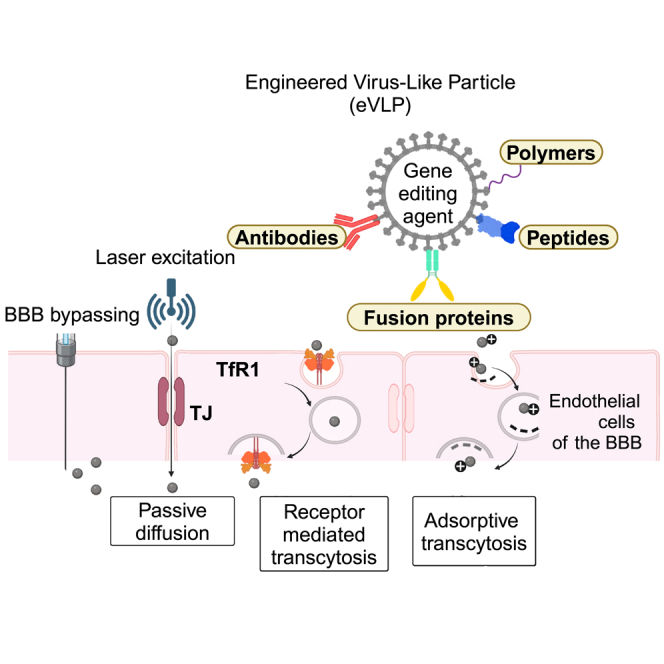

## Teaser

Hewitt and colleagues highlight the engineered virus-like particles as a promising drug delivery platform alternative to viral vectors utilized for *in vivo* gene editing. Several studies have examined pseudotyping delivery vectors to modulate their tissue tropism, and, in this review, the authors discuss the strategies to advance eVLP neurotropism.

## Introduction

Neurological diseases are an extremely heterogeneous group of disorders affecting the CNS. They encompass almost half of all rare diseases and about 80% have a genetic basis.^1^ In recent years, progress in both fundamental and translational research has helped to elucidate details on the molecular signaling and genetic regulation of neurological disorders.^2^ Such advances have broadened the knowledge about the genetic basis of human diseases and revealed that far more disorders are genetic in origin than was previously anticipated.^3^ Recent technological advances such as whole-genome sequencing and whole-exome sequencing have enabled widespread identification of (CNS) disorders with a genetic etiology, with new causative mutations described for Parkinson’s disease, Alzheimer’s disease (AD), Huntington’s disease,^4^^,^^5^^,^^6^ and neuronal ceroid lipofuscinoses (NCLs or Batten disease).^7^^,^^8^ The socioeconomic burden related to these neurological diseases is escalating and is expected to continue globally. Therefore, efficient measures to counter this global health challenge are urgently needed.^9^ Given the structural complexity of the brain and the blood-brain barrier (BBB), the development of therapeutics to target the brain is challenging and hence many investigational drug developments have failed to translate into clinical application. Therefore, to attain the pharmacological success of gene-editing therapeutics, it is important to consider a selection of appropriate drug delivery systems for targeting and penetration of the BBB, drug administration route, and therapeutic index of the drug.^10^

Gene-editing technologies, such as base editing^11^^,^^12^ and prime editing,^13^ have increased in prominence as some of them have already shown success in clinical trials. As the new gene-editor variants are being developed,^14^^,^^15^^,^^16^^,^^17^ many other pre-clinical and clinical trials in treating certain diseases, such as for blood disorders,^18^ cancers,^19^ liver disorders,^20^ amyotrophic lateral sclerosis,^21^ Duchenne muscular dystrophy,^22^ spinal muscular atrophy,^23^^,^^24^ and neurological disorders such as Huntington’s Disease,^25^ genetic epilepsies,^26^ Parkinson’s disease,^27^ and Alzheimer’s^28^ are underway.^29^ To attain their full therapeutic potential, gene-editing agents are required to be encapsulated, protected from degradation, and to bind and traverse the target cell membrane to efficiently deliver the genetic cargo to the nucleus.^30^^,^^31^^,^^32^^,^^33^ They can be packaged as plasmid DNA or as purified proteins or ribonucleoproteins (RNPs),^33^ and the appropriate selection of the delivery system is crucial for the successful therapeutic outcome. Currently, delivery of gene-editing therapeutics is primarily based on viral vectors, lipid nanoparticles (LNPs), and extracellular vesicles. Currently, delivery of gene-editing therapeutics is primarily based on viral vectors, LNPs, and extracellular vesicles.^33^^,^^34^^,^^35^^,^^36^

In this review, we will first examine the characteristics of viral and virus-like delivery systems and then discuss the strategies to attain neurotropism in engineered virus-like particles.

## Viral Vectors

Due to their infectious nature and ability to introduce genes into the host cells, viral vectors are by far the most broadly used method to deliver therapeutic gene therapeutics. A viral vector consists of a protein capsid and/or envelope, transgene, and the regulatory cassette. To package the gene-editing macromolecules, the genome sequences necessary for viral replication and capsid production are removed and replaced. Expression of the transgenes is controlled by the constituent or regulated promoters and other regulatory elements encoded in the viral genome. However, some viruses are limited in their ability to efficiently deliver gene-editing agents due to constraints in their packaging capacity. In the current gene-editing landscape, adeno-associated viruses (AAVs) and lentiviruses (LVs) are at the forefront of pre-clinical and clinical applications.^37^

## AAV Delivery

AAV is a 20- to 25-nm, non-enveloped virus with a cargo capacity of ∼4.7 kb.^36^^,^^38^ Due to the relatively low immunological properties, efficient transduction, and transgene expression in a wide variety of tissues, AAVs have been broadly used for several gene therapies and have shown success in both pre-clinical and clinical settings. Approval of AAV-based gene therapies Luxturna (voretigene neparvovec-rzyl) to treat Leber congenital amaurosis and Zolgensma (onasemnogene abeparvovec) to treat spinal muscular atrophy has marked a milestone in the field of gene therapy.^39^^,^^40^^,^^41^^,^^42^^,^^43^

AAV serotypes identified from humans and non-human primates (NHPs) have been harnessed to deliver CRISPR-Cas9 complexes into different tissues.^44^^,^^45^ These AAV serotypes differ in their tropism based on the changes in the hypervariable regions of the capsid proteins. For example, AAV8 has been shown to efficiently transduce the liver tissue,^20^ while AAV9 transduces a variety of tissues, including muscle,^22^^,^^23^^,^^24^ retina,^30^ heart,^46^ and lung^47^ in mice. Such variations in tissue tropism are primarily attributed to the surface topology of receptor-binding proteins.^44^^,^^48^ Wild-type (WT) AAV serotypes have a low therapeutic index and therefore potentially heighten the risk of immunogenicity and toxicity. Moreover, their performance is likely to be impacted by the pre-existing AAV immunity within the host.^49^ To address these challenges, several modifications to the gene regulatory elements and vector capsid modifications have been thoroughly researched.

The WT AAV genome consists of three open reading frames (ORFs): rep, cap, and X genes, of which the rep gene encodes four proteins that mediate regulation, replication, and assembly and the cap encodes three overlapping capsid proteins: VP1 (90 kDa), VP2 (72 kDa), and VP3 (60 kDa). The icosahedral AAV capsid is a combination of approximately 50 copies of VP3, five copies of VP2, and five copies of VP1. The viral capsid sequence is a key determinant of the tropism and therefore the tropism modifications are often achieved by the chemical and genetic modifications to the capsid. These capsid-modification technologies are discussed in detail elsewhere.^49^^,^^50^

### Modifications to the AAV capsid

To better pursue their therapeutic potential, several improvements have been made to the native AAV capsids in such a way as to interact with a repertoire of cellular receptors to mediate infections. As such, AAV capsids have been chemically or genetically modified to produce hybrid capsids to incorporate a variety of tropism properties. These techniques include, but are not limited to, capsid pseudotyping, directed evolution, rational mutagenesis, or peptide insertions to promote novel receptor-binding activity.^51^

#### Genetic modification of the capsid

Insertion of peptides into the common VP3 region is one such way to genetically modify the virus capsid. For example, I-453, I-587, I-588, I-584, and I-585 sites located within the common VP3 region are susceptible to insertions of targeting ligands. As such, the I-587 site of AAV2 capsid is known to accept peptide insertions up to a size of 34 amino acids and thus characterize AAV2 with different tissue tropism,^49^ whereas genetic modification of VP1 and VP2 introduces complementary split-intein domains to the N terminus of the AAV2 VP2 capsid protein, as well as to the targeting ligands. The modified capsid becomes accessible to form covalent bonds with ligands.^52^

High-throughput selection screening to identify targeting ligands is yet another efficient approach to identifying the modifiable capsid positions and presence of peptides or protein motifs. As such, random oligonucleotide sequence insertions into the cap ORF at sites corresponding to the top of VR-VIII or -IV generate a pool of diverse mutants.^49^^,^^53^ Capsid engineering is also achieved through site-directed mutagenesis of surface-exposed tyrosine (Y), serine (S), threonine (T), and lysine (K) residues.

Mutagenized Y residues of Y444F, Y500F, and Y730F to phenylalanine (F) residues promote the transduction efficacy of AAV2 in the cultured human cells more than that of their WT counterpart.^54^ Enhanced tropisms across various tissue types were further observed with the S residue mutants (S458V, S492V, S662V), T residue mutants (T455V, T491V, T550V, T659V),^55^ and K residues (K490E, K544E, and K549E).^56^

#### Chemical modification of the capsid

Chemical modification of the capsids is an alternative technique to genetic manipulation. It involves modifications to the amino acid composition of the capsid and conjugation of functionalized peptides.^57^ Some examples include re-directed tissue tropisms of glycated AAV2 capsids^58^ and 4-azidophenyl glyoxal (APGO) functionalized rAAV6 capsids.^59^

Mével et al. investigated chemical conjugation of a ligand by the formation of a thiourea functionality between the amino group of the capsid proteins and the reactive isothiocyanate motif incorporated into the ligand. When combined with a hepatocyte targeting ligands such as GalNAc, modified vectors resulted in a higher transduction efficiency in cultured hepatocytes.^57^ Overall, capsid-modification techniques are of great interest and significantly broaden the scope of gene-based therapies.

### Neurotropism of AAV vectors

#### Engineered AAV capsids

Over the past few decades, significant research has been conducted on capsid engineering of AAV to achieve neurotropism.^60^ As such, new clades of primate AAVs have sparked greater interest due to their distinct transduction patterns within the nervous system. For example, rAAV9 is one such serotype that transduces neuronal and glial cell types in the CNS of murine neonates following intravenous injections, which is important for CNS diseases that require intervention during the earlier ages.^61^ The intravenous administration of AAV9 to the neonates and adult mice of amyotrophic lateral sclerosis (ALS) had shown an efficient transgene expression in the neonate brain, dorsal root ganglia and spinal cord motor neurons, and astrocytes,^62^ whereas rAAVrh.8 has been shown to transduce glial cells and neurons located within the cortex, caudate-putamen, hippocampus, corpus callosum and substantia nigra.^61^ Different recombinant AAVs such as rAAVrh.10, rAAV1, rAAV6, and rAAV6.2 have varying transduction profiles across different CNS cell populations. After traversing the BBB, rAAV1 primarily transduces granule cells in the cerebellum, rAAV6, and rAAV6.2 transduce Purkinje cells, while rAAVrh.10, rAAV9, rAAV7, and rAAVrh.39 efficiently transduce motor neurons in mouse neonates.^63^

Other modified capsids of rAAV2-retro and AAV2g9 have been shown to acquire retrograde functionality in projection neurons and also achieve neurotropism derived from both parental counterparts, AAV2 and AAV9.^64^^,^^65^ Moreover, point mutations introduced into the heparin-binding domain of AAV-DJ/8 capsid have been shown to successfully transduce the mouse brain.^66^^,^^67^ Other CNS variants of AAV-PHP.B and AAV-PHP.eB have been shown to cross the BBB and transduce astrocytes, neurons, oligodendrocytes, cerebellar Purkinje cells, and several interneuron populations more effectively than the AAV9.^60^^,^^68^^,^^69^ AAV-PHP.B vectors gain cellular entry by interacting with the LY6A receptors on BBB and therefore their transduction is restricted to species that express the LY6A receptor. The LY6A protein does not have a known homolog in primates and therefore it is unlikely to translate into NHPs.^70^^,^^71^^,^^72^^,^^73^

BI-hTFR1 is another recent example of virus capsid engineering achieved through screening an AAV9-based NNK (NNK refers to a codon sequence where "K" represents either guanine (G) or thymine (T)) capsid library for selective binding to human TfR1 receptors. This AAV9-based NNK capsid library consists of variants with random 7-mer insertions between 588 and 589 VP1 residues. The modified BI-hTFR1 delivered *GBA1* robustly and increased glucocerebrosidase activity across multiple regions of humanized *TFRC* knockin mouse brains. Interestingly, viral transduction within the brain regions implicated in Parkinson’s disease pathology suggests a promising treatment option.^74^

## LV-Mediated Delivery

LV is an enveloped retrovirus with two sense-RNA strands that are bound by nucleocapsid proteins. The LV genome consists of nine genes, including the gag and env. The gag gene encodes the capsid and matrix proteins, while the env gene encodes transmembrane and envelope glycoproteins.^75^ To render the lentiviral vectors non-pathogenic and replication deficient, all virulence genes are removed.^76^ These replication-defective LVs have been utilized in several clinical applications,^77^ such as *ex vivo* genetic modification of beta-thalassemia, X-linked adrenoleukodystrophy (ALD), and metachromatic leukodystrophy.^78^^,^^79^ Moreover, hematopoietic stem cell (HSC) transplantation of LV-transduced CD34+ cells in two X-linked ALD patients showed reverse cerebral demyelination, alleviation of neurological symptoms, and prolonged therapeutic gene expression in treated patients.^78^ Lovotibeglogene autotemcel is a one-time gene therapy that has been evaluated to treat sickle cell disease (SCD). It involves transplantation of autologous CD34+ stem cells transduced *ex vivo* with the LentiGlobin BB305 lentiviral vector encoding a modified beta-globin gene (βA-T87Q-globin) (ClinicalTrials.gov: NCT02140554).

The higher packaging capacity of LVs has been utilized to package bulk and multiplexed genome-editing tools such as CRISPR/Cas9, reporter genes, and single guide RNAs (sgRNAs).^77^ These all-in-one lentiviral CRISPR/Cas9 systems have been shown to transduce and sustain efficient genetic manipulations in a broad range of cell lines including the progenitor and primary cells, which are challenging to transfect.^77^ The tropism of LVs can be modulated by pseudotyping the virions with glycoproteins derived from different enveloped viruses with known tropism.^80^

### Neurotropism of lentiviral vectors

The interactions between the envelope glycoproteins and the cell surface receptor on the target cells allow viral entry. Lentiviral vectors could be targeted to specific brain regions or cell sub-populations by pseudotyping the virus with different envelope glycoproteins^81^^,^^82^ (see Table 1).

**Table 1:** Surface-engineered lentiviral vectors for brain-targeted drug delivery

Brain region	VSV-G	MuLV	Mokola	LCMV	CHIKVG	VEEV	RRV-G	RV-G	RabiesFuG/B2	Reference
**Forebrain**										

Striatum	HIV-1/EIAV	HIV-1	HIV-1/EIAV	EIAV/FIV	HIV-1	HIV-1	–	EIAV/HIV-1	–	Mazarakis et al.,^81^ Won et al.,^82^ Desmaris et al.,^83^ Eleftheriadou et al.,^84^ Kato et al.,^85^ Watso et al.,^86^ Miletic et al.,^87^ Stein et al.,^88^ Trabalza et al.^89^
Thalamus	HIV-1	–	HIV-1	HIV-1	–	HIV-1	–	EIAV/HIV-1	–	Mazarakis et al.,^81^ Kato et al.,^85^ Watson et al.,^86^ Trabalza et al.^89^
Hypothalamus	–	–	–	–	–	–	–	EIAV	–	Mazarakis et al.^81^
Subthalamic nucleus	–	–	–	–	–	–	–	EIAV	–	Mazarakis et al.^81^
Amygdala	–	–	–	–	–	–	–	EIAV	–	Mazarakis et al.^81^
External capsule	HIV-1	HIV-1	HIV-1	–	–	–	–	–	–	Desmaris et al.,^83^ Watson et al.^86^
Olfactive neuron bodies	HIV-1	–	HIV-1	FIV	–	–	–	–	–	Desmaris et al.,^83^ Stein et al.^88^
Mitral cells in the olfactory bulb	–	–	–	–	–	HIV-1	–	–	–	Trabalza et al.^89^
Olfactory tubercles	–	–	HIV-1	–	–	–	–	–	–	Cannon et al.^90^
Globus pallidus	EIAV	–	–	–	–	–	–	EIAV	–	Wong et al.^82^
Subventricular zone (SVZ)	–	–	–	FIV	–	–	–	–	–	Stein et al.^88^

**Midbrain**										

Ventral midbrain	–	–	–	–	–	–	–	HIV-1	–	Kato et al.^85^
Hippocampal dentate gyrus	–	HIV-1	–	–	HIV-1	–	–	–	–	Eleftheriadou et al.,^84^ Watson et al.^86^
Substantia nigra	HIV	–	–	–	–	–	–	–	–	Cannon et al.^90^
Substantia nigra pars compacta	–	–	–	–	–	HIV-1	–	EIAV	–	Mazarakis et al.,^81^ Trabalza et al.^89^
Substantia nigra pars reticulata	EIAV	–	–	–	–	–	–	–	–	Wong et al.^82^
Hippocampus	–	–	HIV-1	–	HIV-1	–	–	–	–	Desmaris et al.^83^
Nigral-dopaminergic neurons	HIV-1	–	EIAV	–	–	–	–	–	–	Desmaris et al.,^83^ Eleftheriadou et al.,^84^ Colin et al.^91^

**Hindbrain**										

Cerebellum	–	–	HIV-1	–	–	–	–	–	–	Colin et al.^91^

**Cell sub-populations**										

Astrocytes	HIV-1	HIV-1	HIV-1	FIV	HIV-1	–	FIV	–	–	Cannon et al.,^90^ Eleftheriadou et al.,^84^ Miletic et al.,^87^ Stein et al.,^88^ Colin et al.,^91^ Kang et al.,^92^ Pertusa et al.^93^
Neurons	FIV/SIVmac	–	HIV-1	–	–	–	–	–	–	Desmaris et al.^83^; Kang et al.^92^; Lieh et al.,^94^
Glial cells	HIV1/EIAV/SIVmac	SIVmac	Bergmann glial cells	–	–	–	SIVmac	–	HIV-1	Mazarakis et al.,^81^ Desmaris et al.,^83^ Miletic et al.,^87^ Colin et al.,^91^ Lieh et al.,^94^ Kato et al.,^95^ Hirano et al.^96^
Brain parenchyma	SIVmac	–	–	–	–	–	–	–	–	Lieh et al.^94^
Cortex	EIAV	–	–	–	–	–	–	EIAV/HIV-1	–	Mazarakis et al.,^81^ Wong et al.,^82^ Kato et al.^85^
White matter	HIV-1	–	HIV-1	HIV-1	–	–		–	–	Watson et al.^86^
Oligodendrocytes	HIV-1	–	HIV-1	–	–	–	FIV	–	–	Watson et al.,^86^ Kang et al.^92^

Lentiviral vectors pseudotyped with various envelope glycoproteins confer an enormous potential for gene therapy of human neurological disease. Primate and non-primate LVs derived from the HIV, feline immunodeficiency virus (FIV), simian immunodeficiency virus, and EIAV are pseudotyped with envelope glycoproteins originated from VSV, rabies virus glycoprotein (RV-G), LCMV, MV, MuLV, Ross River virus glycoprotein (RRV-G), Chikungunya virus glycoprotein (CHIKV-G), Venezuelan equine encephalitis virus glycoprotein (VEEV-G), and fusion glycoprotein B type (FuG-B) or a variant of FuG-B (FuG-B2). Recent pre-clinical studies utilizing these pseudotyped vectors have demonstrated modified neurotropism within different hosts of rats, mice, and monkeys.

In a previous study, Desmaris and co-workers assessed the neurotropism of lentivrial vectors pseudotyped with lyssavirus envelope glycoproteins and injected into the striatum region of the mouse. After 4 weeks from the initial administration, it was observed that Mokola-G pseudotyped lentiviral vectors achieved a robust β-glucuronidase expression compared to the vesicular stomatitis virus G protein (VSV-G) pseudotyped lentiviral vectors.^83^ In another study, astrocyte-specific LV-viral vectors were developed to target gene expression in both neurons and neuronal astrocytes. To further improve the broad distribution of lentiviral vectors they combined retrograde transport properties to target the brain.^6^ The astrocytic gene expression of the lentiviral vectors was achieved by pseudotyping with VSV-G envelope glycoproteins and as well as using promoters such as H1 polymerase III and G1B3 polymerase II, which are known to be highly active in astrocytes. VSV-G envelope glycoprotein interacts with low-density lipoprotein (LDL) receptors located on neurons and astrocytes to mediate endocytosis of the virus.^97^

Biodistribution of lentiviral vectors can be altered using a chimeric fusion glycoprotein variant B2 (FuG/B2) envelope glycoprotein that is characterized by retrograde transport properties. Injecting into the striatum of mice leads to strong transduction in the cortex, amygdala, thalamus, and substantia nigra, indicating potential for enhancing brain therapeutic interventions in the future.^6^ Another similar study showed that lentiviral vectors pseudotyped with rabies virus glycoprotein (RV-G) can promote retrograde axonal gene transfer and efficient neuronal transductions in the CNS. Interestingly, it was observed that RV-G-pseudotyped equine infectious anemia virus (EIAV) results in higher transduction within dopaminergic neurons of the substantia nigra pars compacta. Overall, pseudotyping lentiviral vectors with RV-G protein has been shown to enhance the transduction at the site of injection as well as the distal neurons.^6^

Improved neurotropism of these viral vectors plays a vital role in the treatment of neurological diseases. In mouse brain afflicted with Parkinson’s disease, there was an improved neurotropism of lentiviral vectors that were pseudotyped with vesicular stomatitis virus (VSV), Mokola virus (MV), lymphocytic choriomeningitis virus (LCMV), or Moloney murine leukemia virus (MMLV) envelope glycoproteins. When the VSV-lentiviral vectors and MV-lentiviral vectors were delivered into the substantia nigra of rats, a robust expression in midbrain neurons was observed while LCMV- and murine leukemia virus (MuLV)-pseudotyped lentiviral vector expressions were exclusive to astrocytes.^90^ Extended tropism studies using lentiviral vectors pseudotyped with chikungunya virus glycoprotein reported a selective astrocytic and cell subpopulation transduction pattern within the CNS.^84^ Therefore, it is apparent that envelope modifications of viral vectors have been useful in targeting specific tissues including the brain.

## Drawbacks of Using Viral Vectors

Despite the therapeutic potential, the effective clinical application of gene-editing technologies remains challenging due to the potential for undesired off-target editing. Several strategies have attempted to reduce or eliminate off-target cleavage, including truncated guide RNA with modified scaffolds containing chemical modifications,^98^ and modified CRISPR effector proteins (Cas9 orthologs, high-fidelity variants).^99^ The efficiency of gene-editing technologies is greatly influenced by both the overall expression level and duration of the gene-editing agents within the cells.^36^ Prolonged expression of AAV-mediated gene editing has the propensity to cause off-target mutations over time. More precise spatiotemporal control of transgene expression can be achieved by incorporating tissue-specific promoters into the AAV gene cassette.^100^

Although AAVs are an excellent *in vivo* delivery platform due to the size limits of the AAV, it is not possible to efficiently package larger gene-editing agents, guide RNA, promoters, and other *cis*-regulatory elements within a single AAV particle. To overcome packaging restrictions, split intein to package the gene editor into two individual AAV vectors has been pursued in recent studies. To be reconstituted to form the full-length gene editor and thereby achieve genome editing, both AAV vectors should be delivered into the same cell, hence the editing efficiency may be decreased.^100^ Therefore, to attain the full therapeutic potential of dual AAVs, a higher dose of AAVs is recommended. However, much higher titers of AAV have been suggested to cause dorsal root ganglion toxicity in NHPs and piglets^60^^,^^101^^,^^102^ and life-threatening events in patients that received AAV-based gene therapy.^60^^,^^103^

Immunogenicity is another hurdle impeding viral vector efficacy, which can also lead to safety concerns. For example, AAV-based gene therapies have been found to elicit capsid-specific T cell responses in some people, which could persist for years, affecting the duration of transgene expression.^104^ Moreover, pre-existing immunity to WT AAV has been shown to induce both humoral and T cell-mediated immunity while reducing successful gene transfer.^105^

Supporting the notion that AAV vectors can be genotoxic, another study reported the incidence of hepatocellular carcinomas and angiosarcomas after AAV gene transfer in a mouse model of mucopolysaccharidosis type VII (MPS VII).^106^ Another 10-year post-gene-therapy study revealed oncogenic genome integration of AAV8/AAV9 in six of nine experimental canine candidates and hence raised the safe clinical application.^107^^,^^108^ Therefore, transient expression of genome-editing agents is crucial, and this is typically accomplished by using non-viral delivery systems.

Moreover, selecting the appropriate model is important for achieving clinical success, yet it can be challenging. As such, pre-clinical outcomes experimented in murine models may not necessarily be translatable to higher-order species. For example, systemic AAV-PHP.B vectors have been challenging to translate to NHPs as the BBB permeability of the vector did not extend to NHPs.^71^^,^^72^ In their study, Hordeaux et al. showed that the neurotropic properties of AAV-PHP.B are limited to C57BL/6J mouse strain and underperforms within the BALB/cJ mouse strain, as well as in non-human primates.^73^

Lentiviral vector is yet another important delivery method reported for its functions in *in vivo* gene therapy applications and stable cell transduction. Examples of how lentiviral vectors can be pseudotyped with different envelope proteins from those native to the neurotropic viruses are discussed above. However, lentiviral vectors have been shown to integrate into the host genome causing adverse effects such as insertional mutagenesis. One such gene therapy study reported that HSCs transduced with the BB305 lentiviral vector for SCD adversely developed acute myeloid leukemia (AML).^109^ Another study utilizing a gamma (γ) retrovirus vector expressing interleukin-2 receptor γ-chain (γc) resulted in vector-induced leukemia through enhancer-mediated mutagenesis in four patients with X-linked severe combined immunodeficiency.^110^

Therefore, to mitigate the potential for insertional mutagenesis, integrase-deficient LVs (IDLVs) were developed by mutating the integrase protein and thereby minimizing the proviral integration.^111^^,^^112^^,^^113^ These IDLVs can be used where short-term expression is required and are currently under evaluation for gene-based therapies and vaccines.^114^

Although the modified virus serotypes are useful to target distinct cell populations, safety drawbacks have prevented certain viral vector-based approaches from being utilized for clinical applications, and, therefore, the search for alternative delivery strategies has been intensified.^35^

## Virus-like Particles

Gene-editing agents can be delivered in different forms such as DNA or mRNA; however, ribonucleoproteins (RNPs) offer critical advantages. Virus-like particles (VLPs) are non-infectious assemblies of viral proteins that have been utilized for vaccine development^115^ and have recently been engineered to deliver gene-editing agents.^35^^,^^116^ As they are derived from existing viral scaffolds, they have combined advantages of both viral and non-viral delivery systems. VLPs can be made from different types of viruses, such as avian sarcoma leukosis virus,^117^ but most reported VLPs are based on retroviruses.^118^ The modular nature of the retroviruses has enabled them to further improve cellular tropism. A recent study showed that MLV VLP-Cas9-sgRNA RNPs mediated efficient genome editing in cell lines, primary cells, and in mice. Moreover, *in vivo* injection of VLPs using retro-orbital injections targeting 4-hydroxyphenylpyruvate dioxygenase (Hpd) resulted in successful editing in mouse hepatocytes of tyrosinemic mice.^118^

## Engineered VLPs

Recently, Banskota and co-workers developed engineered VLPs (eVLPs) using an MMLV retrovirus scaffold to encapsulate Cas9 nuclease or base editor ribonucleoproteins (RNPs) and examined the gene-editing potential.^33^^,^^35^ They fused the base editor to the C terminus of the Friend murine leukemia virus (FMLV) gag polyprotein via a linker peptide. When cultured cells were transduced at a higher dose, V1-VLPs mediated a higher editing efficiency. However, single intravenous injections into mice resulted in a low editing of 2% in hepatocytes.^33^^,^^35^

To harness the eVLP platform as a delivery system, several structural arrangements were made. As such, improvements were made with a strong focus on cargo packaging, cargo release, nuclear localization, and component stoichiometry. Subsequently, to improve the cargo release upon eVLP maturation, protease-cleavable peptide linkers (TSTLLMENSS) were added to the second-generation engineered V2.4 eVLPs.^35^

To further improve the potential of eVLPs, nuclear export signals (NESs) and nuclear localization signals (NLSs) were added to the opposite sites of the cleavable linkers. This optimal design to the V3.4 eVLPs resulted in improved cytoplasmic cargo localization in eVLP producer cells while retaining nuclear cargo localization in the transduced target cells.^35^

**Figure 1:**
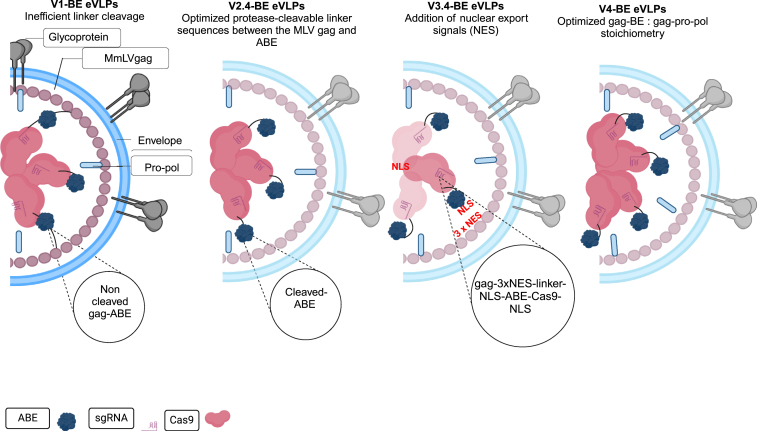
Systemic structural improvement strategies of eVLPs Systemic structural improvement strategies of eVLPs to improve cargo release, cargo packaging, and localization and component stoichiometry. Four generations of BE-eVLPs were produced by optimizing a cleavable linker sequence to improve efficient cargo (denine base editor [ABE]) release upon particle maturation (v2 BE-eVLPs), then nuclear export signals (NESs) were added to enhance cytoplasmic cargo localization to produce v3 BE-eVLPs and component stoichiometry of gag-BE: gag-pro-pol was performed to generate V4 BE-eVLPs. Redesigned from Banskota et al.^35^ Created with BioRender.com.

Additional optimizations to balance the V4 eVLPs structural proteins and cargo component stoichiometry were shown to further increase the protein delivery potency into mammalian cells both *in vitro* and *in vivo* (see Figure 1). Collectively, these improvements resulted in 5- to 26-fold editing efficiencies in contrast to first-generation V1 VLPs. As such, V4 eVLPs mediated editing in various cell cultures and different tissues in mice. This is an improvement over V1 VLPs that was only explicitly determined in the liver (a 26-fold increase). Overall, V4 eVLPs offer a safe therapeutic delivery platform for both base editing^35^ and prime editing agents^116^ and has the opportunity to modulate cell tropism to improve the targeted delivery of therapeutics.^33^^,^^35^

### Neurotropism of eVLPs

It has been suggested that eVLPs can be functionally pseudotyped with different viral envelope glycoproteins to alter their tropism. It has been suggested that these eVLPs can be pseudotyped with different viral envelope glycoproteins to alter their tropism. They pseudotyped V4 BE-eVLPs with neurotrophic FuG-B2 envelope glycoprotein, which had previously been used to pseudotype lentiviral vectors. This glycoprotein contains the extracellular and transmembrane domains of the RV-G envelope glycoprotein and the cytoplasmic domain of VSV-G. When the cultured mouse neuro-2a cells were transduced with FuG-B2-V4 BE-eVLPs, it resulted in efficient transduction and editing, thereby confirming altered tropism.^35^

Overall, these observations establish the potential to advance the neurotropism of eVLPs for brain drug delivery.

## Advancing the Neurotropism of eVLPs to Target and Cross the BBB

While the modification of capsids/envelopes of AAVs and LVs can be adapted to modulate the neurotropism of eVLPs, this section focuses on alternative approaches that could be considered to develop eVLPs with enhanced neurotropism. The BBB acts as an anatomical and physiological barrier that maintains homeostasis in the CNS. The BBB strictly regulates the passage of nutrients and metabolites, thereby protecting the CNS from harmful toxins, pathogens, injury, and disease. It is composed of brain endothelial cells (BECs), a capillary basement membrane (BM), pericytes, and astrocytic foot processes. Tight junctions located between the endothelial cells inhibit the paracellular diffusion of polar molecules, macromolecules, and cells.^119^

When developing eVLPs to target brain drug delivery, it is important to consider the following parameters^120^:

(1) Physiochemical modifications of the therapeutics to target transport across the BBB
(2) Transient biophysical disruption to the BBB to allow transport across the BBB
(3) Alternative routes to administer the drug

Physiochemical modifications of the therapeutics to target transport across the BBB.

There are two routes by which small molecules may cross the BBB:

(1) Paracellular pathway
(2) Transcellular pathway (transcytosis)

The paracellular pathway involves translocating the molecules between BECs and it appears to be exclusively a passive process that is regulated by the tight junctions between endothelial cells. Transcytosis translocates molecules actively or passively through the luminal side of the BEC membrane, traversing the BEC cytoplasm, and then passing across the basolateral side of the BEC into the interstitium of the brain. Passive diffusion of molecules across BECs is dependent on factors such as lipophilicity, electrical charge, and molecular weight. Macromolecules with high molecular weight and hydrophilicity, such as VLPs, are inclined to utilize two main active mechanisms^121^:

(1) Receptor-mediated transcytosis (RMT) (see Table 2)

**Table 2:** Receptor-mediated delivery of therapeutics across the BBB

BBB resident receptors	Receptor targeting strategy
IR	anti-IR antibodies: murine mAb, 83–14 mAbs to the HIR; Boado et al.^122^
TfR	anti-TfR antibodies: murine mAb, OX26 to the rat TfR; Lee et al.^123^ rat mAbs, 8D3 and RI7-217, to the mouse TfR; Lee et al.^123^ murine mAb, 128.1 to the human TfR; Walus et al.^124^ fusion proteins: anti-rat TfR IgG3-Av to deliver biotinylated molecules across the BBB; Penichet et al.^125^
LDLR	fusion protein: targeting the LDLR by fusion of 38 amino acids from the ApoB protein to a therapeutic protein; Masliah et al.^126^ fusogen: G protein of VSV-G; Strebinger et al.^127^
LRP1	angiopep-2: a 19-amino-acid-long oligopeptide that binds to the LRP1; Demeule et al.^128^
Sodium-dependent glutathione transporters	glutathione: enable active transport through sodium-dependent glutathione transporters Reginald-Opara et al.^129^
Nicotinic Ach nAChR receptor	fusion protein: targeting nAChR by fusion of 29 amino acids from the RV-G; Kumar et al.^130^ peptides: a synthetic peptide, KC2S derived from toxin b of the king cobra; Zhan et al.^131^
Endothelial cell growth factor receptor-2 peptides	cell-penetrating peptide: CAYGRKKRRQRRR peptide sequence of TAT-B can induce internalization of molecules with high molecular weights in HBMECs; Cooper et al.^132^
Luminal alpha(2,3)-sialoglycoprotein receptor	sdAbs FC5: llama single-domain antibodies; Muruganandam et al.^133^
NT receptors NTSR1 and NTSR2	46.1 antibody: interact with NT receptors expressed on brain cells (NTSR1 and NTSR2); Georgieva et al.^134^
Other	MiniAp-4: shorter peptidomimetic analog of apamin, renowned for higher BBB permeability via the receptor-mediated transcytosis mechanism; Oller-Salvia et al.^135^

Resident receptors on the BBB have been the target of numerous studies aiming to deliver drugs to the brain. This table provides a summary of strategies used to target these receptors, including the IR, TfR, LDLR, LRP1, sodium-dependent glutathione transporters, nicotinic Ach nAChR receptor, endothelial cell growth factor receptor-2 peptides, luminal alpha (2,3)-sialoglycoprotein receptor, and NT receptors NTSR1 and NTSR2.
(2) Adsorptive-mediated transcytosis (AMT)

In RMT, the delivery of macromolecules is based on the interactions between the ligands and specific receptors on BECs. The receptor and the ligand complex are then packaged into a vesicle through endocytosis and subsequently transported across the BEC cytoplasm. Once they reach the basolateral membrane vesicles, they are bound to it and eventually exocytosed, whereas AMT involves interactions between the cationic molecules and cell membrane binding sites, which leads to endocytosis and subsequent vesicular transcytosis.^120^

The following section focuses on receptor-mediated strategies that can potentially be utilized to advance the neurotropism of the eVLPs. To facilitate direct comparisons with eVLPs, we have also considered examples from VLPs, LNPs, and liposome modifications and how they have resulted in internalization via transferrin receptor (TfR) RMT.

## RMT

Several receptors, including the insulin receptor (IR), TfR, melanotransferrin receptor (MTfR), LDL receptor-related protein 1 (LRP1), and the folate receptor (FR), are known to undergo RMT at the BBB. These endogenous receptors located on the luminal membrane of the brain capillary endothelium are crucial for the uptake of larger molecules such as insulin, leptin, and iron transferrin (Tf).^136^ Of these BBB receptors, TfR1 has been one of the primary targets investigated for RMT.

### TfR

#### Transcytosis of Tf via TfR 1

TfR 1 (TfR1) is highly expressed in the BBB endothelium and has been extensively researched for its potential for brain drug delivery.^136^ However, to utilize the TfR1 for brain drug delivery, it is important to understand the process of TfR-mediated iron uptake at the BBB. In physiological conditions, plasma iron is bound to Tf (holo-Tf), which has shown a high binding affinity to the TfR. Both the ligand and the receptor complex are internalized via clathrin-mediated endocytosis. Acidification of the endosome (a pH drops to ∼5.5) results in conformational changes in Tf directing to iron release. Particularly holo-Tf dissociates and becomes apo-Tf and eventually the endocytic vesicle is recycled back to the plasma membrane in search of more free iron.^137^ Antibodies directed against the TfR are found to promote therapeutic delivery across the BBB and into the brain parenchyma.^138^ Further examples of developing therapeutics in the context of antibodies against TfR1, fusion proteins, and fusogens are discussed below.

### Anti-TfR antibodies

Several strategies have been investigated to utilize antibodies as a carrier for the delivery of drugs across the BBB. Such an anti-TfR antibody system takes advantage of the high abundance of TfR1 on BBB and their ability to shuttle molecules across the BBB. Examples include species-selective rat (OX26)^139^^,^^140^ and mouse (Ri7, 8D3)^141^^,^^142^ antibodies binding to the TfR and transporting the therapeutics into the brain parenchyma. Bioengineering has enabled the design of distinct antibody types; immunoglobulin G (IgG)-like and non-IgG-like antibodies, whose binding properties, size, and half-life can be further modulated.^143^

#### Anti-human TfR antibody

TfR1-binding antibody therapeutics include Pabinafusp alfa, a TfR monoclonal antibody (mAb)-iduronate-2-sulfatase fused to an anti-human TfR antibody (JR 141) for neuropathic mucopolysaccharidosis II. Clinical studies involving JR 141 have been shown to ameliorate heparan sulfate (HS) levels in cerebrospinal fluid (CSF) neurodegeneration in patients with neuropathic mucopolysaccharidosis II (ClinicalTrials.gov: NCT04573023).^144^

Another study reported the re-engineering of erythropoietin (EPO) as a potential brain drug delivery target via the TfRs. Although the native form of EPO does not cross the BBB, research has indicated that the fusion protein of cTfRmAb-EPO can mediate its penetration into the brain. As such, fusion of a 166-amino-acid EPO to the carboxyl terminus of the heavy chain of a chimeric mAb against the mouse TfR resulted in elevated levels of brain uptake and pharmacologic increases in exogenous EPO in the mouse brain following the systemic injection.^145^ Moreover, an enhanced uptake of rsCD4 across the rodent and primate BBB has been shown after conjugation to anti-TfR antibodies 128.1 mAb.^124^

### TfR targeting engineered antibody fragments

In recent years, smaller recombinant antibody fragments such as monovalent antibody fragments Fab and scFv have emerged as alternative targeting domains.^146^ For example, in 2020, Denali Therapeutics reported a BBB transport vehicle (TV) technology, which utilizes an engineered Fc polypeptide that binds to hTfR.^147^ The Denali team has shown significant therapeutic effects with the TV-based technology, successfully targeting lysosomal enzyme iduronate 2-sulfatase (IDS) DNL310 (ClinicalTrials.gov:NCT05371613) to treat Hunter syndrome (or MPS II)^148^ and N-sulfoglucosamine sulfohydrolase (SGSH) DNL126 to treat neuropathic and systemic forms of the Sanfilippo syndrome A (ClinicalTrials.gov:NCT06181136).

Another TV-based enzyme replacement therapy was developed to treat frontotemporal dementia (GRN-FTD) caused by the *GRN* mutations. In a murine model of GRN-FTD, Progranulin-conjugated protein transport vehicles (PGRN-PTV) restored the deficit levels of the lysosomal protein PGRN and was shown to rescue various Grn−/− pathologies, including microgliosis, lipofuscinosis, and neuronal damage.^149^

Brainshuttle (BS) by Roche is another antibody therapeutic developed for AD, which is currently being investigated in the phase I/II trial (ClinicalTrials.gov: 04639050). The BS-mAb module involves the delivery of an anti-amyloid beta (Aβ) antibody fused to a single-chain IgG antigen-binding fragment (fab).^150^

### IR

#### IR targeting antibodies and engineered antibody fragments

Similar to the TfR targeting, the IR expressed at the BBB could be utilized as a target for RMT-based brain drug delivery. Due to the low serum half-life and reduced survival capacity in the blood, insulin is unlikely to complement brain drug delivery. Antibodies recognizing the IR have been developed, such as murine 83-14 mAb to the human IR (HIR), which is a promising brain drug targeting vector that could be used in humans.^151^

Another example includes modifications to the lysosomal enzyme palmitoyl protein thioesterase (PPT1) as IgG enzyme-fusion protein (AGT-194). The IgG domain of this fusion protein is an mAb targeting a BBB transporter receptor transporter such as the IR. The clinical application of AGT-194 has been reported to penetrate the BBB and deliver into the parenchyma, alleviating neuropathologies.^152^

Boado et al. reported on an enzyme replacement therapy (ERT) for mucopolysaccharidosis type I (MPS-I, Hurler’s syndrome). HIR mAb-IDU was developed by fusing human α-L-iduronidase (IDUA) to the carboxyl terminus of the heavy chain of a chimeric mAb to the human IR. A side-by-side comparison was performed to evaluate the BBB penetration of IDUA alone and the HIRmAb-IDUA fusion protein *in vivo*. The higher uptake of the fusion protein suggested HIRmAb-IDU as a potential ERT therapy.^122^

Functionalizing gene editor-eVLPs with antibodies designed against the endogenous BBB receptors could potentially open new prospects to deliver gene-based therapies into the brain. However, it is essential to understand the mechanisms and limitations involved in designing antibodies. As such, binding affinity and valency of the anti-TfR antibodies appear to be a key determinant of their BBB traversing characteristics.

### Fundamental aspects relevant to eVLPs-antibodies

Once bound to the TfR from the blood, vectors are internalized through endocytosis, and the resulting vesicles are forwarded to one of three different pathways, such as being transcytosed into the CNS, trafficked into the lysosome, or recycled back to the apical cell surface.^153^ Therefore, to ensure the targeted delivery of therapeutics, it is important to recognize and correlate where improvements would be necessary. As such, evaluating the affinity of antibodies, their TfR binding mode, and antibody density will be useful to determine their transcytosis capacity.

(1) Bi-specific antibody format

Re-engineering of the therapeutic antibody requires the engineering of a bi-specific antibody (BSA). One arm of the antibody is required to be bound to the receptor (i.e.,; TfR) while the other arm is utilized to attach to the therapeutic target. Compared to the unmodified, bi-specific antibodies have been shown to have a higher brain penetrant ability and to be more uniformly distributed within the brain.^154^^,^^155^

(2) Affinity of antibodies

Antibodies’ affinity for the receptor has an important influence on their ability to dissociate from the receptors and be released into the brain. In 2011, Yu and co-workers reported that anti-TfR antibodies with high affinity to TfR remained associated with the BBB and prevented the antibody-drug conjugates (ADCs) from releasing into the BBB endothelium. Conversely, the reduction of affinity enhanced the RMT of the anti-TfR antibody across the BBB and mediated a broad parenchymal distribution 24 h after dosing. Therefore, to deliver an amount deemed therapeutically useful, it is important to consider that the antibodies are at their low-affinity mode to induce transcytosis and also to disassociate from TfR once it reaches the brain side.^156^

Using Tf-conjugated nanoparticles, Wiley et al. demonstrated a similar impact on nanoparticle delivery at the BBB. It was evident that nanoparticles (∼80 nm in diameter) with a higher avidity were restricted by BBB endothelium, while the high systemic dosing of low-avidity nanoparticles (∼80 nm in diameter) was shown achieve a higher brain uptake (avidity 0.89 nM optimal dissociation constant (KD) to TfR) and subsequently into the brain parenchyma.^157^

(3) TfR binding mode

The TfR-binding mode of an antibody fragment to the TfR is another important aspect of the transcytosis capacity. To determine the monovalent versus bivalent engagement of the TfR, the ADCs were prepared to contain either one or two anti-TfR Fabs conjugated to an anti-β-amyloid mAb and tested in an *in vitro* BBB model. It was shown that the bivalent binding promoted their trafficking into the lysosomes and thus prevented the transcytosis while monovalents successfully entered the CNS. Therefore, the binding mode to the TfR is crucial for successful transport of antibodies across the BBB.^158^

Findings from a recent study involving gold nanoparticles (AuNPs) also indicated the importance of antibody valency contributing to the higher parenchymal accumulation of AuNPs of >70 nm.^159^

(4) Antibody density

Johnsen et al. rationalized that the ligand density contributes to the efficient brain uptake of TfR-targeted AuNPs and chemotherapeutic drug-loaded liposomes. To make comparisons, both AuNPs and liposomes were functionalized with the same density of rat anti-mouse TfR antibody, RI7217 per surface area of the particle and potential for further increase in brain uptake was evaluated using ligand densities ranging from 0.15, 0.3, and 0.6 × 10^3^ antibodies/μm^2^. Interestingly, AuNPs functionalized with the highest density were shown to achieve an enhanced accumulation in brain capillaries and were transported into the murine brain parenchyma.^160^

In contrast, achieving high affinity to RMT receptors may be optimal for certain carrier antibodies, such as those targeting IRs, to enhance therapeutic delivery mechanisms. Therapeutic delivery using anti-IR has been investigated for delivering enzymes and therapeutic antibodies.^161^ HIR mAb-IDUA fusion (valanafusp alpha) is one such therapeutic protein for Hurler syndrome caused by a deficiency of the enzyme IDUA. It combines an HIR mAb with IDUA. The HIR antibody maintains high-affinity binding to IR, resulting in increased IDUA enzymatic activity.^162^ Another pre-clinical *in vivo* study on developing mAbs targeting the Abeta amyloid peptide in AD showed that high-affinity HIR binding of HIR mAb-anti-amyloid resulted in a 10-fold increase in brain uptake compared to the anti-amyloid antibody controls.^163^

## Antibodies or Engineered Antibody Fragments to Target Other BBB Receptors

### LDL receptors

The LDL receptor (LDLR) expressed in brain capillary endothelial cells mediates the transport of lipoproteins and a diverse array of other ligands across the BBB via RMT. LDLR-targeted drug delivery can be achieved by conjugating a protein(s) with high affinity to the LDLR. One such example is the application of the apolipoprotein B (ApoB)-LDLR binding domain approach for the development of CNS-penetrating peptides for AD. This involved targeting the LDLR by the fusion of 38 amino acids from the ApoB protein to a therapeutic protein. When combined with the LDLR binding domain, a significant brain penetration at the BBB was observed.^126^

Therefore, to engineer cell trafficking properties, eVLPs can be potentially modified with cell-penetrating ligands such as N-methyl-D-aspartate (NMDA) receptor peptides, nicotinic acetylcholine receptor (nAChR) peptides, and *trans*-acting activation transduction (TAT) peptide.

### nAChR-targeting peptides

A 29-amino-acid peptide derived from the RV-G fused to a sequence of nine arginine residues is suggested to drive an absorptive-mediated transport across the BBB. This peptide effectively targets the nicotinic acetylcholine receptor (nAChR) of which the expression is restricted to astrocytes and neurons.^130^

KC2S is a synthetic peptide derived from toxin b of the king cobra (*Ophiophagus hannah*) that has a high binding affinity with nAChRs. In cultured brain capillary endothelial cells, KC2S-linked micelles were taken up and delivered systemically, while the dye-loaded KC2S-micelles were observed to accumulate slowly in the brain, and drug-loaded KC2S-micelles provided a modest survival benefit in an orthotopic glioma model. In their study, Zhan et al. further showed that loop 2 of *O. hannah* toxin b binds with neuronal nAChRs and enhances intracranial drug delivery in mice.^131^

### Growth factor receptor-2-targeting peptides

HIV-derived *trans*-activating regulatory protein (TAT) is a key activator of HIV-1 transcription. This 86- to 101-amino-acid protein has been shown to penetrate BECs in both *in vitro* and *in vivo* models. Cooper and co-workers showed that the CAYGRKKRRQRRR peptide sequence of TAT-B can induce internalization of molecules with high molecular weights in human brain microvascular endothelial cells (HBMECs).^132^ TAT is known to bind to several cell surface receptors, including vascular endothelial cell growth factor receptor-2 on BECs.^164^ *In vivo* studies resulted in BBB penetration of intravenously administered TAT peptide in the hippocampus, occipital cortex, and hypothalamus of the mouse brain.^85^ Therefore, peptide-mediated BBB penetration has important implications for future therapeutic developments.^164^

### Luminal alpha (2,3)-sialoglycoprotein receptor-targeting antibody FC5

Single-domain antibodies (sdAbs) have been investigated for creating bi-functional proteins or bi-specific antibodies. Such characteristics offer several benefits compared to the current peptide and antibody-based methods. One such novel single-domain llama antibody, FC5, transmigrates across the BBB and is initiated by interacting with cell surface α (2,3)-sialoglycoprotein and then internalized via clathrin-coated endocytic vesicles. Both *in vitro* and *in vivo* studies have shown the ability of FC5 to cross the intact BBB and, hence, its potential for FC5-drug conjugates brain drug delivery strategies.^133^

### NTSR1 and NTSR2 receptor targeting antibody 46.1

Georgieva et al. recently identified the antibody 46.1, which was generated following screening of a human single-chain Fv (ScFv) phage library. Following the intravenous administration, antibody 46.1 was observed to accumulate in the post-vascular brain. To further evaluate the brain delivery properties, the scFv-Fc form of the antibody was fused with a 13-amino-acid peptide neurotensin (NT) payload. Intravenously administered 46.1-scFv-Fc-LL-NTScFv 46.1 mediated the transport of NT across the BBB and accumulated in the median preoptic nucleus and striatum. Overall, the 46.1 antibody is capable of transporting drug cargo into the CNS.^134^

## BBB traversing peptide shuttles and analogs

Peptides are another successful strategy to traverse the BBB and have been shown to have advantages over mAbs. Anand et al. developed a bi-functional nanocontainer Tat (FAM)P22-MVIIA using cell-penetrating HIV-Tat modified *Salmonella typhimurium* bacteriophage P22 capsids. When tested *in vitro*, these modified VLPs were efficiently taken up by the RBMVEC-BBB model via an endocytic pathway.^165^

To accommodate the brain delivery, Pang et al. designed a virus-like particle/RNAi nanocomplex modified with an apolipoprotein E (ApoE) peptide. The ApoE has an inherent affinity for the LDLR on the BBB. When tested *in vivo*, the modified virus-like particle/RNAi nano complexes (VLP/RNAi) (diameter of 30 nm) were observed to penetrate the BBB and ameliorate malignant brain tumors in a mouse model of glioblastomas (GBMs).^166^

Moreover, peptides derived from venoms have demonstrated the potential as BBB penetrating peptide shuttles. However, given the cytotoxic nature, venoms in their crude stage cannot be used for therapeutic applications. Such development of minimized versions of chlorotoxin (CTX) (MinCTX-3) and apamin (MiniAp-4) peptide toxins has shown the potential for brain-targeted drug delivery.^167^

### MiniAp-4

Apamin is a bicyclic 18-mer peptide derived from *Apis mellifera* bee venom and has a higher BBB permeability. However, the therapeutic use of apamin is restricted due to higher toxicity, immunogenicity, and structural complexity. MiniAp-4 is a shorter peptidomimetic analog of apamin, renowned for higher BBB permeability via the receptor-mediated transcytosis mechanism. MiniAp-4 has been shown to carry covalently bound macromolecules and nanoparticles. As such, 12-nm AuNPs cargoes tethered with MiniAp-4 have been shown to actively cross the BBB and deliver cargo into the brain parenchyma of mice.^135^

### MiniCTX-3

CTX is a 36-amino-acid peptide neurotoxin isolated from the venom of the giant yellow Israeli scorpion *Leiurus quinquestriatus* and a synthetic chlorotoxin (TM-601) is currently utilized to deliver anti-cancer therapeutics for glioma.^168^ MiniCTX-3 is a synthetic peptide based on CTX peptide that is protease resistant. It has been shown to transport both small molecules as well as AuNPs of 12-nm diameter in a human BBB cellular model.^169^

Given their higher BBB permeability and stability and BBB-traversing ability, these peptide shuttles could potentially be utilized to improve the neurotropism of eVLPs.

### Fusogens

Tropism of viruses, VLPs, and engineered virus-like particle delivery systems can be modulated via pseudotyping strategies. This involves the fusion of a viral protein (or fusogen) on the surface of the vector particle. Pseudotyping allows the viral membrane to fuse with the target host cell membrane receptors, thereby releasing the cargo into the cytoplasm of the target cell. G protein of vesicular stomatitis virus (VSV-G) is one such popular glycoprotein that interacts with LDLRs and possibly other LDLR family members and hence the broad range of cell tropism. Several other viral envelope proteins or their analogs have been investigated for their different tropism characteristics and it is important to engineer them to optimize their performance.^127^

Recently, Strebinger et al. described a technique known as delivery to intended recipient cells through envelope design (DIRECTED) to program viral vector tropism via receptor-antibody interactions. It involves cell fusion components of both natural and engineered origin and includes various cell-targeting strategies. Once the cells are transduced, interactions between the glycoprotein and cognate cell receptor promote cell targeting and entry. Certain fusogen membrane fusions are pH dependent and independent of the engagement of a cellular receptor. Once they have gained entry, the endosomal process results in successful payload delivery. To redirect the vector tropism, they developed strategies such as using a chimeric antibody-binding protein or an SNAP-mediated covalent linkage (SNAP-tag) to recruit or immobilize antibodies on the viral envelope. Overall abundance of cell surface receptors, endocytosis of target receptor, endosomal formation, and pH-dependent conformational changes are important. Due to the programmable and compatibility nature of DIRECTED particles, it can be applied for enveloped delivery systems such as lentiviral particles, eVLPs, and Cre recombinase VLPs (CreVLPs) and has been anticipated to enhance the applicability across various cells including the brain.^127^

As has been reported for VLPs,^118^ we reason that the use of multiple glycoproteins to pseudotype eVLPs could overcome receptor saturation and therefore realize the combined benefits of different glycoprotein tropisms.

## Surface Engineering Using Polymers

VLP surface can be engineered by introducing surface-accessible amino acids to provide conjugation sites for the synthesis of non-metallic nanomaterials via covalent bonding. Examples include but are not limited to VLP derivatives based on tobacco mosaic virus (TMV) carboxylic acid moiety of aspartate and glutamate reactions with amines, which have been functionalized with biotin, chromophores, and crown ethers while Y can conjugate with poly(ethylene glycol) (PEG).^170^ We envisage modifications such as polymer coating of eVLPs may prove valuable to improve their brain-targeted delivery.

### PEG coating

The capacity to achieve brain penetration with larger vectors is important for the clinical translation of therapeutics. Given the success achieved so far with nanoparticles of similar diameter (100–150 nm), there is potential for improving eVLPs through PEGylation to enhance brain delivery. Compared to the non-polymer-coated nanoparticles, the polymer-coated nanoparticles show an improvement in transport across the BBB and accumulation in the brain. Nance et al. demonstrated that nanoparticles as large as 114 nm in diameter can diffuse through the human brain cortex tissue and in the rat brain but only when they are densely coated with PEG. The proof of concept was pre-clinically evaluated using paclitaxel-polymer-coated nanoparticles. This study also highlighted the importance of the surface density of PEG on a nanoparticle. When quantified using nuclear magnetic resonance (NMR)-based methods, it was estimated that a 100-nm nanoparticles should be PEGlyated with about nine PEG molecules (molecular weight = 5 kDa) per 100 nm^2^ of particle surface.^171^

Additionally, cationic bovine serum albumin (CBSA)-conjugated PEG-poly(d,l-lactide-co-glycolide) (PLGA) nanoparticles (CBSA-NPs) (diameter of ∼100 nm) indicated a higher biodistribution in the brain tissue. A dosage of 60 mg/kg CBSA-NP in mice caudal vein demonstrated a higher accumulation of CBSA-NP in the lateral ventricle, third ventricle, and periventricular region compared to the control.^172^ Moreover, PEGylated nanoparticles (Maleimide-PEG3500-PLA40000 and methoxyPEG2600-PLA40000) conjugated with OX26 mAbs also resulted in a robust gene expression throughout the CNS, including neurons, choroid plexus epithelium, and the brain microvasculature of the monkey brain.^173^

## Magnetic targeting for BBB delivery

Magnetic targeting of nanoparticles is an alternative approach that enables them to reach deep parenchymal targets. This involves the application of an external magnetic field (EMF), thereby guiding the pathway to the nanoparticles across the BBB. The EMF can be exerted either by the magnets implanted intracranially or placed outside the skull of mice. Systemic administration of polystyrene nanospheres with trapped magnetic nanoparticles (MNPs) of ∼100 nm were shown to accumulate in the brain parenchyma. Although this technique could open new prospects for brain-targeted drug delivery, damage to the brain cells induced by the magnetic field is yet to be evaluated.^174^

Overall, current techniques on brain-targeted delivery provide insights into the further development of eVLPs. We have considered examples of VLPs, LNPs, and liposome modifications that can hamper the applications of the strategies into clinically useful eVLPs.

## Transient biophysical disruption to the BBB to allow transport across the BBB

To improve drug delivery through the paracellular pathway, efforts have been made to alter the properties of the BBB. As such, Li and co-workers demonstrated a reversible modulation of the BBB by laser stimulation.^175^ Transcranial picosecond laser stimulation of AuNPs enhanced the BBB permeability and AuNPs were diffused through the tight junction complementing the paracellular pathway. AuNPs were modified by antibody BV11 to specifically target junctional adherence molecules (JAMs),^176^ which are highly expressed in brain vascular endothelial cells. They reported that the BBB permeability changes are completely reversible and do not lead to significant disruption in the spontaneous vasomotion or the structure of the neurovascular unit. Subcutaneous injections of AuNPs are functionalized by anti-JAM-A antibody BV11 on the scalp and then a surgical procedure is performed to peel the scalp to expose the skull to apply laser through the intact skull. It allowed the entry of immunoglobulins, viral vectors, and liposomes and suggested opening new avenues for brain drug delivery applications.^175^ Hence, we anticipate that a similar strategy could be considered to improve the brain target delivery of eVLPs.

## Alternative Routes to Bypass the BBB

### Barrier bypass delivery methods

Different techniques, such as intranasal (IN) delivery, intrathecal (IT) delivery, and interstitial delivery, are currently being used to bypass the BBB, and more information on these strategies has been reviewed in detail elsewhere.^120^

### IN delivery

The IN cavity is a highly vascular absorptive surface located at a closer proximity to the brain. Intranasally administered drugs may pass through the olfactory epithelium and/or nasal epithelium and subsequently enter the systemic vasculature to be absorbed by lymphatics or continue to pass through a paracellular route into the neurons. The IN route has been widely used in veterinary clinical neurology applications, such as to deliver therapies for seizures.^120^

In addition, endocytosis of the drug by olfactory sensory neurons or trigeminal nerve endings within the nasal mucosa has also been shown to mediate axonal transport of the drug through the olfactory of trigeminal nerve pathways. Repeated transsynaptic processes within these neurons promote robust distribution of the drug into different brain regions. Moreover, intranasally administered drugs avoid the first-pass hepatic effect and hence sustain a higher bioavailability of the drug in the CNS. Furthermore, IN delivery offers practical advantages such as a non-invasive nature, not requiring any expertise to administer the drug, and reduced adverse effects.^120^

### IT delivery

IT administration is a well-established drug delivery technique that has been widely used to treat both humans and animals.^177^ It involves direct administration of therapeutics into the CSF that flows through the thecal sac.^178^ Examples include the administration of analgesia or therapeutics to treat spasticity (via baclofen pump)^179^ and CNS neoplasms.^178^ IT delivery can also be achieved through intracisternal magna (ICM) or intracerebroventricular (ICV) injection, by which the CSF can be accessed. The ICV administration of drugs using an implanted device such as the Ommaya reservoir has been widely used to treat pediatric and adult patients to treat CNS disorders.^178^ For example, cerliponase alfa, or recombinant human tripeptidyl peptidase 1 (TPP1), is a US Food and Drug Administration (FDA)-approved ERT infused through an Ommaya reservoir to treat children with CLN2 Batten disease.^180^^,^^181^ Advantages of IT injections include consistent surgical procedures, reduced dosage, and smaller drug volumes. However, risks associated with the invasive technical approaches raise safety concerns. As with all surgical manipulations, IT injections are also associated with disadvantages such as the potential for backflow, damage to the brain tissue, and variable distribution beyond the injection site. Distribution of the therapeutics is majorly dependent on the diffusion rate of the drugs into the brain cells from the injection site and hence the drug concentration is decreased logarithmically.^10^ It can also be technically challenging in different species, given their differences in size, age, and anatomical limitations.^177^^,^^178^

### Interstitial delivery

Although the IN and IT routes bypass the BBB, direct administration of small molecules and macromolecular compounds into the interstitium is considered the most direct delivery route. This technique often achieves higher therapeutic drug concentrations in the brain and poses a reduced systemic drug exposure.^120^

Direct interstitial delivery can be performed by a parenchymal bolus injection, implantation of biocompatible and biodegradable materials, and convection-enhanced delivery (CED). CED is another strategy known for homogeneous and efficient brain drug delivery. Classically, a small hydrostatic pressure gradient built from the syringe pump drives the distribution of the drug to bypass the BBB. CED has been shown to improve the biodistribution of AAVs in the brains of large animals and humans.^182^

For example, the AAV2-aromatic l-amino acid decarboxylase (AADC) enzyme, which catalyzes the conversion of levodopa (primary drug) to dopamine, was delivered via CED to the bilateral putamen to safely increase the AADC levels in Parkinson's disease patients (ClinicalTrials.gov: NCT03065192). More recently, Stahl and co-workers observed robust editing in the mouse striatum mediated by Cas9 RNPs and AAVs delivered via CED. They showed that bilateral CED injections of engineered cell-penetrant 4x-Cas9-2x NLS fusion protein improved the efficient delivery of Cas9 RNPs within the mouse brain. However, adverse effects such as fluid backflow, white matter edema, and formation of air bubbles have been reported relating to CED, highlighting the importance of improved brain drug delivery systems.^183^^,^^184^

However, similar to other invasive techniques, interstitial delivery has its drawbacks. As such, its need for repeated administrations, and specifically the bolus injections, may cause uncontrolled spatial distribution of drugs in the interstitium that could potentially cause reflux along the injection tract or expose non-target areas of the brain.^120^^,^^185^

## Safety considerations with VLPs

The clinical success of the eVLPs entails their ability to bind and deliver the gene-editing agent to the target cells. Access to the target cells and endosomal escape within the cells is achieved by surface modifications or specific targeting moieties derived from different viral envelopes. However, higher concentrations of viral envelope proteins are cytotoxic and therefore using non-viral proteins to promote VLP cell entry and endosomal escape is encouraged.^186^ It has also been implicated that vectors pseudotyped with certain virus envelope glycoproteins could potentially be susceptible to inactivation by the human serum complement. For example, VSV-G-pseudotyped lentiviral vectors generated in human cell lines have been found to be inactivated either by restricted vector membrane incorporation or by functional blockage by human cell membrane complement control proteins such as CD-55 or CD-59. This observation highlights the importance of developing vectors with amphotropic envelopes for *in vivo* applications.^187^

Improved particle survival and circulation could be achieved by re-engineering the eVLP surface. Previously, Milani and co-workers produced lentiviral vector particles lacking polymorphic class-I major histocompatibility complexes that inhibited human primary T cell activation. Lentiviral vector surface modification was achieved by inactivating beta-2 microglobulin (B2M) genes in producer cells.^188^ Pre-existing immune responses to viral envelope proteins such as VSV-G may contribute to eliciting an immunogenic response within the host.^189^

## Summary

There is an imperative need for therapeutic intervention to improve brain-targeted drug delivery, but efficient drug penetration is limited by the tightly regulated BBB. There have been extensive efforts to overcome brain drug delivery challenges and recent advances in gene-editing technology are useful in treatments and translational neuroscience.^3^^,^^190^ Due to the structural complexity of the brain and the BBB, gene therapy vectors are being injected directly into regions of disease pathology through IT, intraventricular, or intravascular routes.^191^ To circumvent the BBB, direct injection into brain parenchyma and intraventricular infusion has been utilized in most of the clinical trials. For example, intraventricular infusion of cerliponase alfa in children with CLN2 disease has proved a clinical success (ClinicalTrials.gov: NCT01907087 and NCT02485899).^192^ However, these invasive delivery techniques have an inherent risk of surgery-related side effects, and, therefore, alternative, less invasive delivery strategies are required.

Alternative delivery platforms such as VLPs have been long studied as a potential drug delivery modality. These VLP particles lack viral genetic material and are therefore considered safer than viral-mediated gene delivery. Recently developed engineered VLPs can be utilized to package and efficiently deliver therapeutic gene-editing proteins and has proved to have minimal off-target effects. They strategically developed the “bottlenecks” of conventional VLPs related to cargo packaging, localization, and cargo release. This fourth-generation eVLP has shown a significant 16 times more cargo packaging capacity as well as a 26-fold increased gene-editing activity within cells and animal models.^35^ eVLPs have a combination of key characteristics of both viral and non-viral delivery and they have been shown to efficiently deliver therapeutic RNPs, avoiding the risk of host genome integration and prolonged expression of therapeutic agents.^33^^,^^35^ In contrast to the recurring challenges associated with the viral and non-viral delivery platforms, eVLPs are likely to have an increased delivery potency in cultured cells as well as efficient delivery in animals.

However, in the process of engineering eVLPs for brain delivery, it is important to consider several factors, such as avoiding relying on naturally existing ligands, evaluating the antibody fusion format, affinity and effector function of the antibody, therapeutic efficacy, biodistribution, and the brain uptake efficiency. RMT of macromolecules is achieved through modifications to the vectors targeting specific transporter or transporters, such as the TfR, LDL receptor-related proteins (LRP-1 and LRP-2), or IR.^193^ Pseudotyping with viral glycoproteins is one such modification that has been successful for re-targeting retroviral Cas9-VLPs to different cell targets in humans or other non-rodent species.^194^ In eVLPs, the scaffold is derived from the MMLV, which is known to be pseudotyped with different envelope glycoproteins.^35^^,^^195^ Similarly, eVLPs can also be pseudotyped with different glycoproteins,^35^^,^^116^ and interest continues in exploring the use of other targeting moieties, such as antibodies or receptor ligands, conjugated to the surface of the eVLPs. The choice of cognate RMT system is an important aspect when designing a BBB delivery vector.^193^ The modifiable nature of the VLP surfaces facilitates the binding of peptides and proteins via chemical conjugation, genetic engineering, or a combination of both techniques.^196^ Typical constituents of the VLP capsid surfaces, such as K, cysteine, glutamic acid, and methionine, can be directed for surface modification with various biomolecules, including proteins, antigens, and small molecules.^197^

Moreover, it is also important to acknowledge the different species-specific RMT receptor expression profiles,^70^ and therefore pre-clinical translation of modified eVLPs should involve comprehensive evaluations across different animal models such as rodents and NHPs. Furthermore, the discovery of novel BBB-restricted RMT targets continues to be imperative. In this regard, recent advancements in RNA sequencing (RNA-seq) and proteomics techniques have proved instrumental in identifying novel CNS-specific RMT targets.^161^

In summary, eVLP-based gene-editing delivery systems offer a safe and promising alternative to existing techniques like viral and non-viral vectors. Moreover, the combined advantages of both viral and non-viral delivery systems and the programmable nature of the eVLP structure make eVLPs adaptable to improve the delivery of genetic cargo. As more CNS target drug delivery strategies are developed, we will gain new insights on how to further improve the precision of eVLPs for gene therapy delivery to the brain.
